# Associations between Genotype–Diet Interactions and Weight Loss—A Systematic Review

**DOI:** 10.3390/nu12092891

**Published:** 2020-09-22

**Authors:** Sandra Bayer, Vincent Winkler, Hans Hauner, Christina Holzapfel

**Affiliations:** 1 Institute for Nutritional Medicine, University Hospital Klinikum rechts der Isar, School of Medicine, Technical University of Munich, 80992 Munich, Germany; sandra.bayer@tum.de (S.B.); vincent.winkler@tum.de (V.W.); hans.hauner@tum.de (H.H.); 2 Else Kröner-Fresenius-Center for Nutritional Medicine, School of Life Sciences, Technical University of Munich, 85354 Freising, Germany

**Keywords:** genetic variant, single nucleotide polymorphism, weight loss, nutrigenomics, dietary intervention, personalized nutrition

## Abstract

Studies on the interactions between single nucleotide polymorphisms (SNPs) and macronutrient consumption on weight loss are rare and heterogeneous. This review aimed to conduct a systematic literature search to investigate genotype–diet interactions on weight loss. Four databases were searched with keywords on genetics, nutrition, and weight loss (PROSPERO: CRD42019139571). Articles in languages other than English and trials investigating special groups (e.g., pregnant women, people with severe diseases) were excluded. In total, 20,542 articles were identified, and, after removal of duplicates and further screening steps, 27 articles were included. Eligible articles were based on eight trials with 91 SNPs in 63 genetic loci. All articles examined the interaction between genotype and macronutrients (carbohydrates, fat, protein) on the extent of weight loss. However, in most cases, the interaction results were not significant and represented single findings that lack replication. The publications most frequently analyzed genotype–fat intake interaction on weight loss. Since the majority of interactions were not significant and not replicated, a final evaluation of the genotype–diet interactions on weight loss was not possible. In conclusion, no evidence was found that genotype–diet interaction is a main determinant of obesity treatment success, but this needs to be addressed in future studies.

## 1. Introduction

In the last four decades, obesity has been identified as one of the major health risks worldwide and has reached pandemic extents [1]. According to the World Health Organization (WHO), over one-third of the world’s population is overweight and 13% are described as obese [2]. Obesity adversely affects almost all physiological functions of the body and increases the risk of developing multiple diseases such as type 2 diabetes, cardiovascular diseases, and certain cancers [3,4]. Overweight and obesity are mainly caused by a long-term positive energy balance as a result of the modern lifestyle which is characterized by low physical activity and high consumption of energy-dense food [5]. To tackle obesity, multiple lifestyle intervention strategies have been developed with limited average success rates. Different diets varying in macronutrient content (e.g., low-fat/low-carb) have been investigated and compared to identify dietary regimes for successful weight loss [6]. The “one size fits all” approach for weight reduction is critically discussed. As a consequence, customized, personalized dietary recommendations are gaining more attention to fit individual needs.

In general, people lose weight to a varying extent under specific diets and this heterogeneity may depend on various factors, e.g., adherence to treatment or genetic factors [7]. The identification of multiple genetic loci associated with body mass index (BMI) and body fat distribution in genome-wide association studies (GWAS) supports the hypothesis of strong genetic interference [8,9,10,11]. A recent GWAS identified 941 BMI-associated genetic loci, which account for approximately 6% of BMI variation [11], and some specific single nucleotide polymorphisms (SNP) have been discussed as being involved in the pathogenesis of obesity [11,12,13]. Frayling et al. demonstrated an additive association between the risk allele of SNP rs9939609 of the fat mass and obesity-associated (*FTO*) gene and higher body weight [14]. Furthermore, the A allele of the SNP rs571312 of the melanocortin-4 receptor (*MC4R*) gene is associated with an increased BMI by 0.23 kg/m^2^ [10].

Previous findings from genetic association studies led to the investigation of the relationship between certain genotypes and the effect of diets on body weight. In the Diet Intervention Examining The Factors Interacting with Treatment Success (DIETFITS) randomized clinical trial, Gardner et al. found that there was no significant difference in weight change between the low-carb and low-fat diet group after 12 months and no diet-genotype interaction for weight loss was found [15]. The Nutrient-Gene Interactions in Human Obesity: Implications for Dietary Guidelines (NUGENOB); Diet, Obesity, and Genes (DiOGenes); and Food4Me trials showed similar results [16,17,18]. The Food4Me trial investigated various stages of tailored nutrition. In comparison to the control group, a personalized dietary recommendation led to significantly greater weight loss [16]. In contrast, personalization based on specific SNPs had no further benefit in this study compared to other strategies of personalization [16]. However, Xiang et al. concluded in their meta-analysis with 6951 participants that *FTO* risk allele carriers (SNP rs9939609) show significantly greater weight loss than non-carriers [19]. This supports the hypothesis that specific genotypes may play a role in weight management [6]. In addition to that, studies have shown substantial inter-individual differences in metabolic response to certain meal challenges [20,21]. These results can be partly explained by genetic variations between the participants. Therefore, there is a growing interest to investigate and to understand genotype-diet interactions. A review by Livingstone et al. investigated the association between certain risk alleles and macronutrient intake [22]. They concluded that these risk alleles play a role in altering the dietary consumption of fat and protein and thus influencing weight loss [22]. The same research group also investigated the relationship between *FTO* minor alleles and weight loss in a meta-analysis [23]. The result indicated that individuals carrying *FTO* minor alleles do not show any significant differences regarding body weight response to a dietary intervention compared to non-carriers [23]. Taken together, the results were inconclusive and substantiate the need for further analyses regarding the association between genotype, diet, and weight loss.

This systematic literature search aimed to investigate whether there are genotype–diet interactions on weight loss. By including interaction terms, this analysis provides new information on the combined effect of SNPs and macronutrients on weight loss. The results may contribute to substantiate genotype-based dietary recommendations for the prevention and treatment of overweight and obesity.

## 2. Materials and Methods

This review was registered in the International Prospective Register for Systematic Reviews (PROSPERO, registration number CRD42019139571) and followed the Preferred Reporting Items for Systematic Review and Meta-Analyses (PRISMA) [24].

### 2.1. Search Strategy

The four electronic databases PubMed, Embase, Web of Science, and Cochrane Library were searched on 2 July 2019 by one person (S.B.). To identify articles examining the research question of this review, the search items were subdivided into three blocks: genetics, nutrition, and weight. For the genetic block, the following search items were used: “single nucleotide polymorphism”, “SNP”, “genotype”, “genetic variant”, and “gene variant”. To include nutritional aspects, we applied the following search items: “energy”, “caloric”, “calorie”, “fat”, “carbohydrate”, “carb”, “diet”, “dietary”, “nutrition”, and “nutritional”. The nutritional item “protein” was not included in the search strategy as it plays a minor role in the treatment of obesity. Search items for the block weight included the following terms: “weight”, “weight loss”, “weight reduction”, “BMI”, and “body mass index”. The search items in each block were combined with the Boolean operator “OR”. The three blocks were then combined with the Boolean operator “AND”. Depending on the database, plural forms of the search items, quotation marks, and/or asterisk were used and filters for language (“English”) and species (“human”) were applied. Reference lists of eligible articles were checked by hand to identify additional articles.

### 2.2. Study Selection

The study selection adhered to the PICO (population, intervention, control, and outcomes) criteria [25]. Studies with the following criteria were included: (a) intervention study, (b) diet described, (c) availability of SNP data, (d) outcome: weight loss, (e) interaction term of genotype x diet. Literature not in English, animal studies, and studies with participants having a severe disease (e.g., cancer) or impaired mobility were excluded. Furthermore, studies in children, pregnant and breastfeeding women, and transplant patients were excluded. Studies with no statistical application term of a genotype–diet interaction on weight loss were excluded as well. Studies with dietary interventions not focused on weight loss or on macronutrients were not considered. Two reviewers (S.B., V.W.) independently screened titles, abstracts, and full texts for eligibility. In cases of discrepant evaluations, a third reviewer (C.H.) assessed the article for eligibility. Reasons for exclusion were documented. If the full text was not available, we contacted the authors. For the screening process, we used the reference management software EndNote X9 (Thomsen Reuters, New York, NY, USA) and Microsoft Excel 2016 (Microsoft Corp, Redmond, WA, USA).

### 2.3. Data Extraction

The data extraction was performed independently by two reviewers (S.B, V.W.) with the software program Microsoft Excel 2016 (Microsoft Corp, Redmond, WA, USA). A third reviewer (C.H.) was consulted if inconsistencies emerged. The following data were extracted: authors, publication year, study name, study design, description of the study population, sample size, measurement of weight, intervention time, description of dietary intervention, assessment of dietary intake, genes of interest, SNP, statistical results, and statistical adjustment procedures.

### 2.4. Reporting Strategy

Due to the expected heterogeneity of the eligible studies, we did not perform a meta-analysis of the data. Studies with statistically significant and not significant genotype–diet interactions were treated equally in this review. Data are presented narratively.

### 2.5. Risk of Bias and Quality Assessment

The risk of bias of randomized controlled trials (RCTs) was assessed by the Cochrane Collaboration’s risk-of-bias tool for randomized trials (RoB 2) [26]. The studies were evaluated for the randomization process, deviations from the intended intervention, missing outcome data, measurement of the outcome, and selection of the reported results. The risk of bias was judged either as low risk, some concerns, or high risk.

Non-randomized intervention studies were examined by the Cochrane Collaboration’s Risk of Bias in Non-Randomized Studies—of Intervention (ROBINS-I) assessment tool [27]. Assessment was performed for confounding, selection of participants into the study, classification of intervention, deviations from intended interventions, missing data, measurement of outcomes, and selection of the reported results. Here, the risk of bias was also judged as low risk, some concerns, or high risk.

For the genotype–diet interaction term, we further applied an assessment tool for the quality of genetic association studies [28]. The validity of associations was assessed by 11 questions focusing on chance, risk, and confounding. Points ranging from −11 to +11 were given to rate quality as follows: rather high (+4 to +11 points), intermediate (−3 to +3 points), or low (−4 to −11 points).

## 3. Results

**Figure 1:**
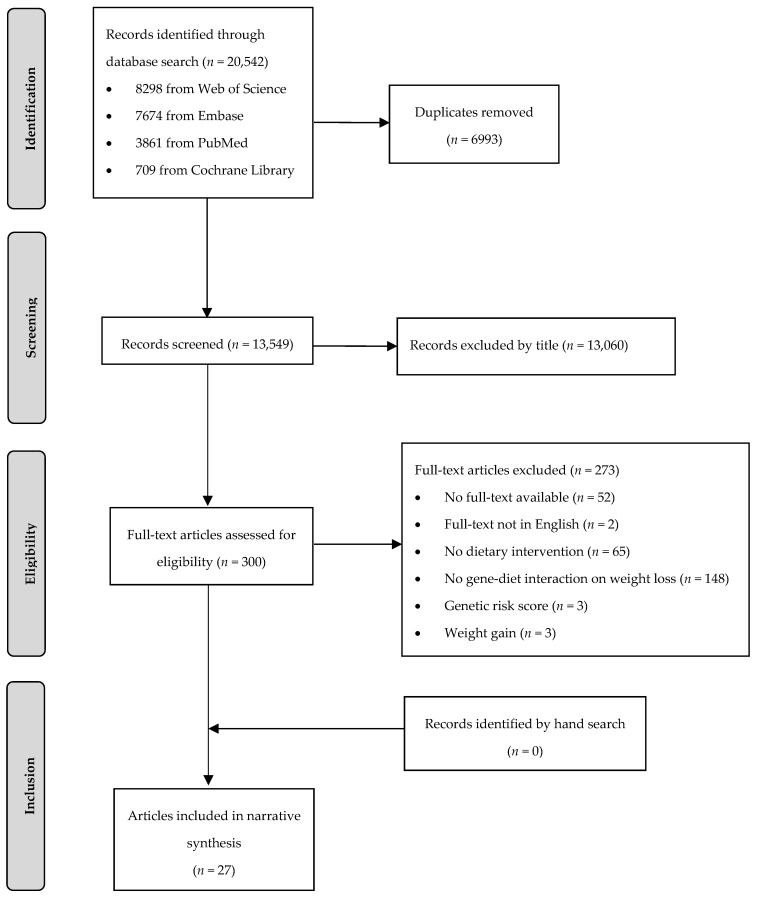
Flow chart of the systematic literature search according to Moher et al. [24].

In total, 20,542 articles were identified, of whom 6993 articles were removed as duplicates (Figure 1). During title and abstract screening, a further 13,249 articles were excluded. Twenty-seven articles (26 publications based on eight RCTs and one publication based on one non-randomized trial) met the PICO criteria and were included in this systematic review.

### 3.1. Characteristics of Studies Included

The eight human intervention trials included in this systematic review are described in Table 1: NUGENOB [17,29,30,31,32,33,34], Development of Nutrigenetic Test for Personalized Prescription of Body Weight Loss Diets (Obekit) [35,36], Preventing Overweight Using Novel Dietary Strategies (POUNDS Lost) [37,38,39,40,41,42,43,44,45,46,47,48,49,50,51], Dietary Intervention Randomized Controlled Trial (DIRECT) [41,45], Prevención con Diet Mediterránea (PREDIMED) [52], DiOGenes [33], one trial from Italy [53], and one trial from Spain [54] (Table 1). The publication time ranged from 2006 to 2019. The sample sizes were from 147 to 7447 participants. The duration of the interventions ranged from 4 weeks to 4 years. For the collection of dietary intake, most articles used 24 h recalls [37,38,39,40,41,42,43,44,45,46,47,48,49,50,51], followed by dietary records [17,29,30,31,32,33,34,35,36], a combination of 24 h recalls and food frequency questionnaires (FFQ) [41,45], as well as other questionnaires [52], or no further information was given [53,54]. The studies differed in characteristics of participants such as ethnicity, age, BMI, and disease status, as well as the kind of dietary intervention (Table 1).

### 3.2. Study Quality and Risk of Bias

**Figure 2:**
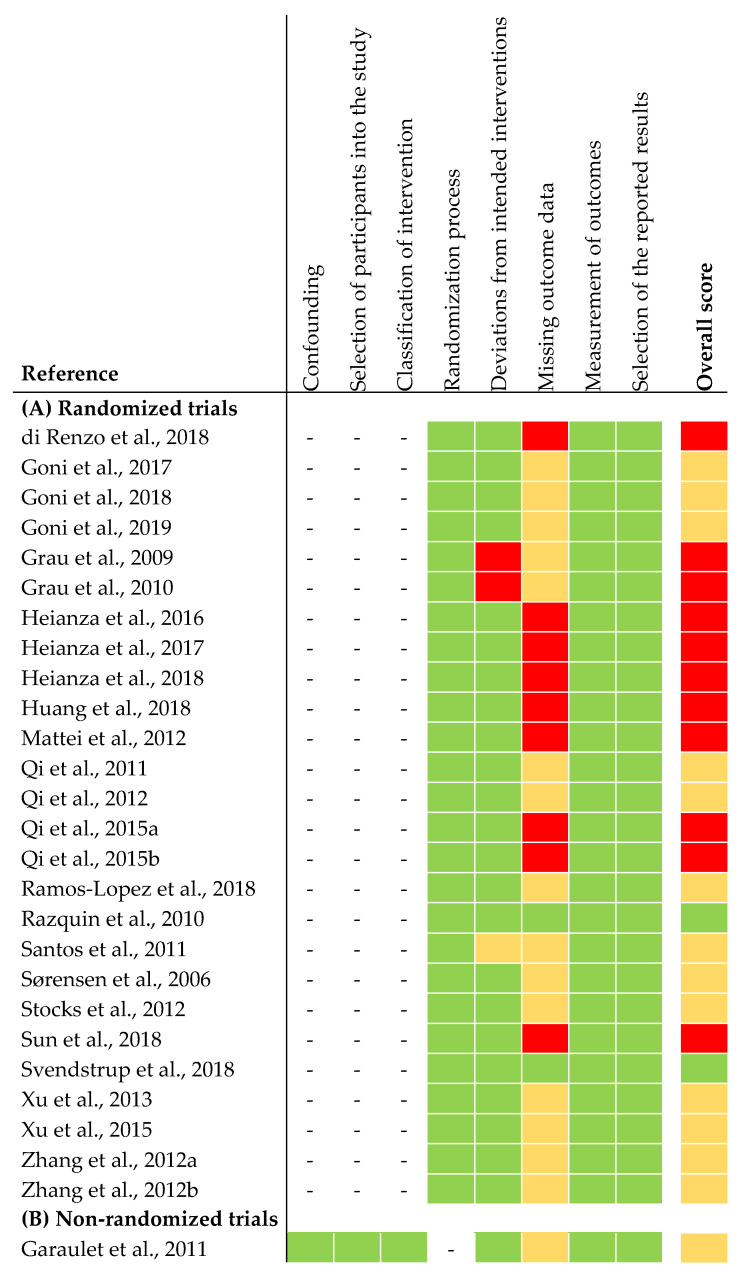
Risk of bias assessment of articles included in narrative synthesis. (**A**) Risk of bias assessment of randomized trials [26]. (**B**) Risk of bias assessment of non-randomized trials [27]. Overall score: the risk of bias was judged as low risk (green), some concerns (yellow), high risk (red).

The risk of bias assessment of the selected studies is shown in Figure 2. In summary, two out of 26 articles from the RCTs were judged to be at low risk of bias for all domains. Thirteen articles were judged to raise some concerns. This was due to high drop-out rates during intervention. Because of missing data on sample size in the intervention groups at the end of intervention, we judged 13 articles to be at high risk of bias. The non-randomized trial was judged to be at moderate risk of bias for all domains. The latter was due to missing information about exclusion criteria of participants during intervention (Figure 2).

**Figure 3:**
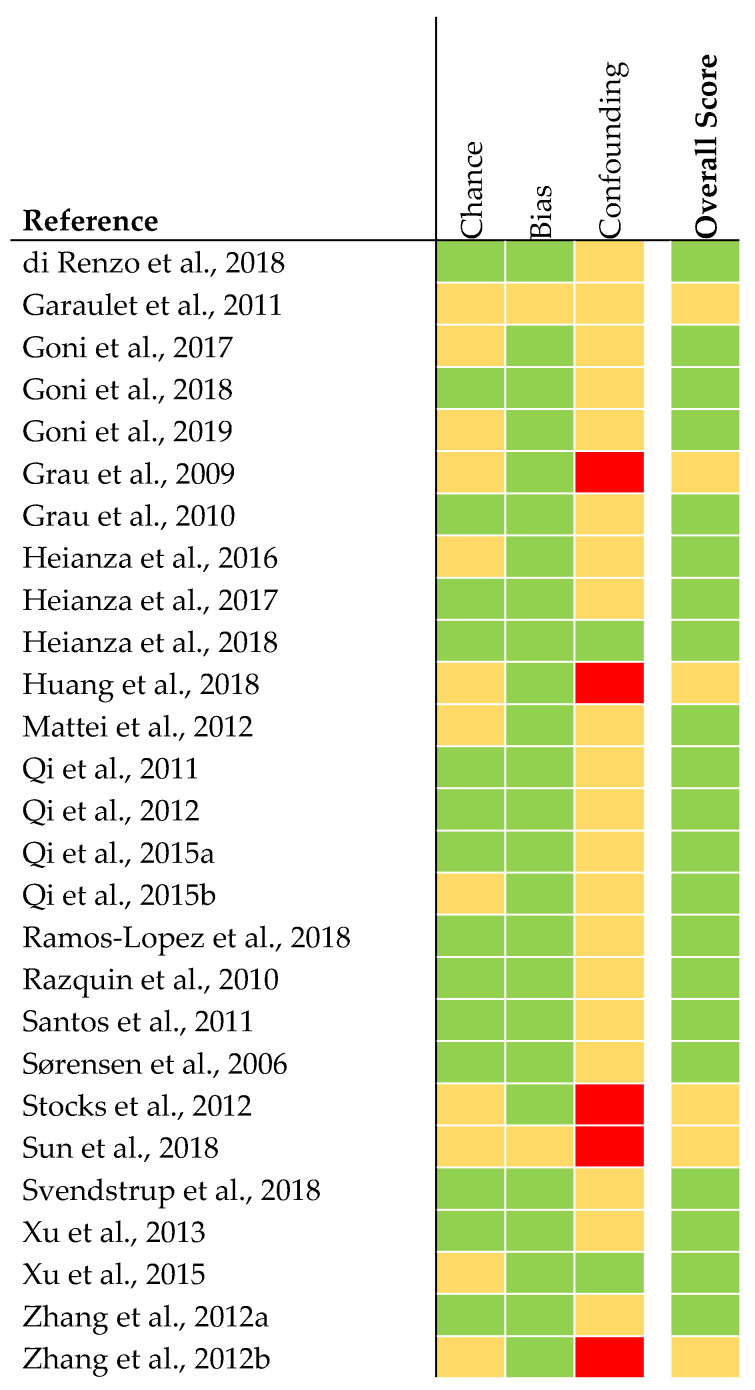
Quality assessment of genetic association studies [28]. The quality was judged as rather high (green), intermediate (yellow), or low (red).

The quality of analyses concerning genetics was judged to be rather high in 21 articles (Figure 3). The quality of six articles was judged as being intermediate. This was due to missing statistical analysis concerning confounding parameters (e.g., adjustment for ethnicity).

### 3.3. Main Findings

#### 3.3.1. Interaction of Genotype and Fat Intake on Weight Loss

In total, an interaction of genotype and fat intake on weight loss was assessed for 60 different genetic loci and 88 different SNPs (Table 2). 

Most of the SNPs (*n* = 80) were analyzed once and six of them (adenylate cyclase 3 (*ADCY3*) SNP rs10182181 [35]; adiponectin, C1Q and collagen domain containing (*ADIPOQ*) SNP rs266729 [32]; tumor necrosis factor alpha (*TNFα*) SNP rs1800629 [32]; cytochrome P450 family 2 subfamily R member (*CYP2R1*) SNP rs10741657 [46]; melatonin receptor 1B (*MTNR1B*) SNP rs10830963 [37]; nuclear factor of activated T cells 2-interacting protein (*NFATC2IP*) SNP rs11150675 [47]) showed a statistically significant interaction with fat intake on weight loss.

SNPs within eight genetic loci (*FTO* SNP rs9939609; HNF1 homeobox A (*HNF1A*) SNP rs7957197; interleukin 6 (*IL6*) SNP rs1800795; hepatic lipase C (*LIPC*) SNPs rs6082, rs1800588, rs2070895; peroxisome proliferator-activated receptor gamma isoform 2 (*PPARG2*) SNP rs1801282; protein phosphatase, Mg2+/Mn2+-dependent 1K (*PPM1K*) SNP rs1440581; transcription factor 7-like 2 (*TCF7L2*) SNPs rs7903146, rs12255372; transcription factor AP-2 beta (*TFAP2B*) SNP rs987237) were examined for the genotype x fat intake interaction on weight loss twice in 12 articles [17,29,30,32,33,41,42,48,49,52,53,54]. No statistically significant interaction between the *FTO* SNP rs9939609 and fat intake on weight loss was seen [30,53]. After 6 months of intervention, a statistically significant interaction between the *HNF1A* SNP rs7957197 and fat intake on weight loss was seen in the POUNDS Lost and the DIRECT trial, as well as in the pooled data [41]. A greater weight loss was observed in participants with the T allele with a high-fat diet compared to those without the T allele. However, after 2 years of intervention, no statistically significant interaction could be found between the *HNF1A* SNP rs7957197 and fat intake on weight loss [41].

The study participants of the PREDIMED [52] and the NUGENOB [32] trials were analyzed to investigate the *IL6* SNP rs1800795 x fat intake interaction on weight loss. In the PREDIMED trial, homozygous carriers of the risk allele showed a greater weight loss with the Mediterranean diet with olive oil supplementation compared to a low-fat diet than heterozygous carriers and non-carriers after 3 years of intervention [52]. This result could not be replicated in a similar analysis from the NUGENOB trial [32]. Here, no statistically significant interaction between the *IL6* SNP rs1800795 and fat intake on weight loss was found after 10 weeks of dietary intervention. Furthermore, there was no statistically significant interaction between the *LIPC* SNPs rs6082, rs1800588 in the NUGENOB trial, and SNP rs2070895 in the POUNDS Lost trial and fat intake on weight loss. The SNPs rs1800588 and rs2070895 are in high linkage disequilibrium (*r*^2^ > 0.8) [32,48]. The results on the *PPARG2* SNP rs1801282 x fat intake on weight loss were controversial. While a study from Spain with 1465 participants found a lower percental weight loss compared to baseline weight in homozygous and heterozygous carriers compared to the non-carriers on the high-fat diet [54], no statistically significant interaction was seen in an analysis on the NUGENOB dataset [32].

While the NUGENOB trial did not reveal a statistically significant interaction between the *PPM1K* SNP rs1440581 and fat intake on weight loss after 10 weeks of intervention [29], the homozygous carriers of this gene variant in the POUNDS Lost trial showed a lower weight loss on a high-fat diet after 6 months as well as after 2 years of intervention compared to the heterozygous and the non-carriers [49]. Contrary results were also found for the *TCF7L2* SNP rs7903146 x fat intake interaction on weight loss. After 10 weeks of intervention, the NUGENOB trial identified a lower weight loss on a high-fat diet in homozygous carriers compared to heterozygous carriers and non-carriers combined [17]. In the POUNDS Lost trial, no statistically significant interaction between the *TCF7L2* SNP rs7903146 x fat intake on weight loss was found [42]. Additionally, no statistically significant interaction was seen between the *TCF7L2* SNP rs12255372 and fat intake on weight loss. The *TCF7L2* SNPs rs7903146 and rs12255372 were in high LD (*r*^2^ > 0.8) [42].

One article examined the interaction of *TFAP2B* SNP rs987237 and fat intake on weight loss in the DiOGenes and NUGENOB trial, respectively [33]. An additive genotype–diet interaction model of the NUGENOB trial showed that homozygotes for the A allele lost more weight with the low-fat than the high-fat diet, whereas homozygotes for the G allele lost more weight on the high-fat diet compared to the low-fat diet. These findings were not confirmed by the DiOGenes trial [33].

#### 3.3.2. Interaction of Genotype and Carbohydrate Intake on Weight Loss

Three articles investigated the interaction of genotype and carbohydrate intake on weight loss (Table 3) [38,42,44]. No significant interactions could be found between the consumption of carbohydrates and the fibroblast growth factor 21 (*FGF21*) SNP rs838147 [38] and the *TCF7L2* SNPs rs7903146 and rs12255372 (LD *r*^2^ > 0.8) on weight loss [42]. Among the participants of the POUNDS Lost trial, a statistically significant interaction between the insulin receptor substrate 1 (*IRS1*) SNP rs2943641 and the highest-carbohydrate diet was found after 6 months of intervention [44]. Homozygous carriers of the risk allele showed greater weight loss on the highest-carbohydrate diet (65 energy%) than heterozygous carriers or non-carriers after 6 months of intervention. After 2 years of intervention, no statistically significant effect of the interaction between *IRS1* SNP rs2943641 and carbohydrate diet on weight loss could be found (Table 3).

#### 3.3.3. Interaction of Genotype and Protein Intake on Weight Loss

In total, seven publications assessed the interaction of genotype and protein intake on weight loss [39,40,42,43,46,49,51] (Table 4). All of them analyzed data from the POUNDS Lost trial and used an additive model for genetic analysis. No significant interactions between the amylase alpha 1A- amylase alpha 2A/B (*AMY1-AMY2*) SNP rs11185098 [40]; *CYR2R1* SNP rs10741657 [46]; 7-dehydrocholesterol reductase (*DHCR7*) SNP rs12785878 [46]; GC vitamin D-binding protein (*GC*) SNP rs2282679 [46]; gastric inhibitory polypeptide receptor (*GIPR*) SNP rs2287019 [43]; lactase (*LCT*) SNP rs4988235 [39]; neuropeptide Y (*NPY*) SNP rs16147 [51]; *PPM1K* SNP rs1440581 [49]; and *TCF7L2* SNPS rs7903146, rs12255372 (LD *r*^2^ > 0.8) [42] and the protein intake on weight loss was found (Table 4).

## 4. Discussion

This systematic review gives an overview of genotype x diet interactions and their association with weight loss. The literature search identified 27 articles in which the interaction of 91 SNPs within 63 genetic loci and fat [17,29,30,31,32,33,34,35,36,37,40,41,42,43,45,46,47,48,49,50,51,52,53,54], carbohydrate [38,42,44], or protein intake [39,40,42,43,46,49,51] on weight loss was investigated.

Most publications (*n* = 24) focused their interaction term on the macronutrient fat. This may be due to the fact that fat is the most energy-dense macronutrient and, therefore, most weight-loss studies have a focus on reducing fat intake. Furthermore, it might be assumed that obesity-associated SNPs play a role in fat intake [60]. However, the results of our systematic review present an inconsistent picture for genotype–fat intake interaction and weight loss. Most findings were not significant and not replicated in other trials. The statistically significant findings were related to 12 SNPs in 12 distinct genetic loci. However, the *ADCY3* SNP rs10182181 [35], *ADIPOQ* SNP rs266729 [32], *CYP2R1* SNP rs10741657 [46], *MTNR1B* SNP rs10830963 [37], *NFATC2IP* SNP rs11150675 [47], and *TNFα* SNP rs1800629 [32] were examined in only one trial each. This lack of replication excludes a robust interpretation of the results.

The general findings of this systematic review are in line with a publication about the association between the genetic variant *FTO* rs9939609, and dietary intake and BMI, indicating no significant interaction between the *FTO* variant and dietary intake on BMI [60].

A genotype–fat intake interaction on weight loss was examined twice with eight SNPs in 12 articles, but the findings showed inconsistent results as in one study a significant interaction between the SNP and fat intake on weight loss was found, whereas this result could not be replicated in the other study [17,29,32,33,42,49,52,54]. This might be explained by the different sample sizes, study durations, or dietary interventions. Furthermore, a statistically significant interaction between the *HNF1A* SNP rs7957197 and a high-fat diet on weight loss could be seen in the POUNDS Lost trial as well as in the DIRECT trial [41]. Carriers of the T allele (minor allele) showed a greater weight loss on a high-fat diet than non-carriers after 6 months of intervention. Variants of the *HNF1A* gene are known to be associated with diabetes [61,62]. Thereby, the risk of developing diabetes might be influenced by the lifestyle and weight status of the individual [41,63]. One possible explanation of the underlying mechanism might be that a high-fat diet downregulates *HNF1A* gene expression in the pancreas [64,65] and thereby might cause weight loss and improvement of insulin resistance [64,65]. However, after 2 years of intervention, no statistically significant interaction was found between the *HNF1A* SNP rs7957197 and fat intake on weight loss in both studies as well as in the pooled data [41]. This result may also be explained by a loss of adherence to the diet and high drop-out rates [56]. Moreover, after 12 months of intervention, the participants in both trials regained body weight [56,58].

Similar inconclusive results were found for the potential interactions of SNPs and both carbohydrate and protein intake and their effect on weight loss. All long-term results reached no statistical significance and were investigated only within the POUNDS Lost trial. Due to the fact that the sample size of the POUNDS Lost trial with 811 participants is low compared to most genetic association studies [10,14], the statistical power to reach significant results was rather limited. Moreover, the high drop-out rates (*n* = 179) and the regain of body weight, as described earlier [56], may further decrease statistical power.

Irrespective of such inconsistencies, this systematic review provides three main findings on the topic of genotype–diet interactions and weight loss. First, there are many “significant” findings observed in single and mostly small studies without replication in others. Second, the number and size of studies to examine genotype–diet interactions on weight loss are rather limited. The 27 publications identified refer to only eight weight loss trials of which 15 publications were based on data from the POUNDS Lost trial and another seven papers analyzed potential interactions in the database of the NUGENOB trial. The third message from this analysis is the considerable heterogeneity of the identified studies. There were substantial differences among the trials not only in the selection of SNPs, but also in study design, dietary interventions, intervention duration, and sample size. In addition, dietary intake data—highly relevant for the research question—were collected using different methods, all of those self-reported and with a high risk of recall and reporting bias, which may further complicate such analyses. It is noteworthy that many intervention studies were excluded because they investigated the association between single SNPs and weight loss without considering any interaction term for genotype x diet on weight loss. This might be explained by the fact that weight loss was not the primary outcome [66] or was due to publication bias, as negative results concerning genetics are commonly not published [67]. To promote the field of genotype–diet interactions on weight loss it is, therefore, crucial to collect and analyze genetic material more frequently or regularly in dietary intervention studies. However, as the treatment of overweight and obesity is a complex process with many factors involved, this request may be a great challenge.

All studies selected for this review are based on a hypothesis-driven approach investigating defined candidate genes. This means, that no hypothesis-free GWAS are available for weight loss intervention trials. It appears likely that other genetic loci rather than the obesity-associated loci may play a role in weight loss and macronutrient intake. Therefore, broader approaches may be needed to overcome the limitations of current studies, e.g., by pooling of RCTs and broader genotyping. The identification of SNPs associated with thinness might be an innovative approach [68].

### Strengths and Limitations

The strength of this systematic review is the inclusion of all SNPs for which a genotype–diet interaction on weight loss was available. The narrative synthesis was based on any nutritional intervention differing in the macronutrient distribution as well as hypocaloric diets, as others mainly focus on other types of trials [19,23]. We assessed the risk of bias as well as the methodological quality of the included articles. A limitation of many publications was that studying the interaction between genetic loci and macronutrient composition of a diet was not the primary aim of the respective study and was usually a post hoc analysis. Due to the high heterogeneity of the SNPs, a confirmation of the findings was not possible in most cases and it is also not possible to perform meta-analyses of the extracted studies. Our review could not identify studies investigating the additive effect of common SNPs in the form of genetic risk scores and specific diets on weight loss. Furthermore, we did not include studies focusing on copy number variants; mutation analysis; haplotypes; and studies investigating the association of genotype–diet interaction and BMI, body fat, or other obesity-related variables.

## 5. Conclusions

This systematic review summarized the results of genotype–diet interactions on weight loss. Independent of the kind of dietary intervention, most of the genotype–diet interactions on weight loss were not significant. The high heterogeneity of the SNPs and the lack of replications does not allow us to draw a final conclusion, as robust data on possible genotype–diet interactions on weight loss are missing. Most findings were based on the POUNDS Lost and NUGENOB trials. Therefore, more studies with larger sample sizes are needed to adequately address this highly relevant question in obesity research.

## Figures and Tables

**Table 1:** Description of the trials.

Study Name	Country, Ethnicity	Study Population	Intervention	Duration of Intervention	Weight Loss in kg (Mean ± SD)	Collection of Dietary Data	Reference
NUGENOB	United Kingdom, Netherlands, France, Spain, Czech Republic, Sweden, Denmark Caucasian	771 participants inclusion: BMI ≥ 30 kg/m^2^; age 20–50 years exclusion: weight change > 3 kg in last 3 months; drug treated hypertension; diabetes mellitus; hyperlipidaemia; untreated thyroid disease; surgically/drug-treated obesity; pregnancy; alcohol/drug abuse; participation in other study.	600 kcal/day less (1) low-fat diet: 20–25 E% fat, 15 E% protein, 60–65 E% carbs (2) high-fat diet: 40–45 E% fat, 15 E% protein, 40–45 E% carbs	10 weeks	(1) −6.9 ± 3.4 (2) −6.6 ± 3.5	Dietary record	[55]
Obekit	Spain: Caucasian Hispanics	147 participants inclusion: BMI 25–40 kg/m^2^; unrelated exclusion: cardiovascular disease; diabetes mellitus treated with insulin; pregnant and lactating women; use of medications that affect body weight; weight change > 3 kg in last 3 months; unstable use of medication for hyperlipidaemia, type 2 diabetes and treatment of hypertension.	30% energy restriction (1) low-fat diet: 22 E% fat, 18 E% protein, 60 E% carbs (2) moderately high protein diet: 30 E% fat, 30 E% protein, 40 E% carbs	16 weeks	(1) −8.1 ± 4.1 (2) −7.6 ± 4.0	Dietary record	[35]
POUNDS Lost	United States: 80% Whites, 15% African Americans, 3% Hispanics, 2% Asians or other	811 participants inclusion: BMI 25–40 kg/m^2^; age 30–70 years exclusion: diabetes mellitus; cardiovascular disease; medications that affect body weight; insufficient motivation.	750 kcal/day less (1) low fat/low-protein diet: 20 E% fat, 15 E% protein, 65 E% carbs (2) low-fat/high-protein diet: 20 E% fat, 25 E% protein, 55 E% carbs (3) high-fat/low-protein diet: 40 E% fat, 15 E% protein, 45 E% carbs (4) high-fat/high-protein diet: 40 E% fat, 25 E% protein, 35 E% carbs	2 years	6 months (1) −6.54 ± 0.42 (2) −6.80 ± 0.42 (3) −6.37 ± 0.42 (4) −6.42 ± 0.42	24 h recall	[56,57]
					2 years (1) −3.26 ± 0.56 (2) −5.03 ± 0.58 (3) −3.87 ± 0.59 (4) −3.98 ± 0.42		
DIRECT	Israel	322 participants inclusion: BMI ≥ 27 kg/m^2^; age 40–65 years; presence of type 2 diabetes or coronary heart disease exclusion: pregnant or lactating women; serum creatinine level ≥ 2 mg/dl; liver dysfunction; gastrointestinal problems; active cancer; participating in another diet trial.	(1) low-fat diet: 1500 kcal women, 1800 kcal men, 30 E% fat, 10 E% saturated fats, 300 mg cholesterol intake (2) Mediterranean diet: 1500 kcal women, 1800 kcal men, no more than 35 E% fat, 30 to 45 g of added olive oil and a handful of nuts (3) low-carbohydrate diet: non-restricted calorie diet, 120 g carbohydrates, based on Atkins diet	2 years	(1) −2.9 ± 4.2 (2) −4.4 ± 6.0 (3) −4.7 ± 6.5	FFQ and 24 h recall	[58]
PREDIMED	Spain: European	7447 participants inclusion: age 55–80 (men)/60–80 (women) years; diabetes or three or more major cardiovascular risk factors exclusion: history of cardiovascular disease; severe chronic illness; drug or alcohol addiction; history of allergy or intolerance to olive oil or nuts; low predicted likelihood of changing dietary habits.	(1) low-fat diet (2) Mediterranean diet + olive oil (3) Mediterranean diet + nuts	4 years	(1) −0.10 ± 0.3 (2) −0.21 ± 0.2 (3) −0.07 ± 3.8	Questionnaire	[52,59]
DiOGenes	Netherlands, Denmark, United Kingdom, Greece, Germany, Spain, Bulgaria, Czech Republic	938 participants inclusion: BMI 27–45 kg/m^2^; age < 65 years exclusion: > 3 kg weight change within 2 months prior to the study; medication; certain disease.	low-calorie diet: Modifast diet, four items per day, one item between 202–218 kcal, 880 kcal, fat 20 E%, carbs 54 E%, protein 26 E%	8 weeks	−11.1 ± 3.5	Dietary record	[18]
No acronym	Italy: Caucasian	300 participants inclusion: Caucasian; Italian; age > 16 years.	(1) control group: general recommendations (2) Mediterranean diet: isocaloric, <25 E% fat, 20 E% protein, 55 E% carbs	4 weeks	(1) TT genotype: −1.27 ± 3.89 A carriers: −0.62 ± 1.26 (2) TT genotype: −3.41 ± 6.47 A carriers: −2.25 ± 11.79	n. a.	[53]
No acronym	Spain	1465 participants inclusion: BMI 25–39.99 kg/m^2^; age 20–65 years exclusion: medication for blood pressure, lowering glucose or lipids; diabetes mellitus; chronic renal failure; hepatic disease; cancer.	600 kcal less; women: 1200–1800 kcal/day; men: 1500–2000 kcal/day; Mediterranean diet: 35 E% fat (<10 E% saturated fats + 20 E% monounsaturated fats), 15–20 E% protein, 50 E% carbs	different between participants	G carriers: −6.84 ± 5.54 CC genotype: −7.35 ± 5.68	n. a.	[54]

BMI, body mass index; carbs, carbohydrates; DiOGenes, Diet, Obesity, and Genes; DIRECT, Dietary Intervention Randomized Controlled Trial; E%, energy%; FFQ, Food Frequency Questionnaire; h, hour; kcal, kilocalories; kg, kilograms; n.a., not available; NUGENOB, Nutrient-Gene Interactions in Human Obesity: Implications for Dietary Guidelines; Obekit, Development of Nutrigenetic Test for Personalized Prescription of Body Weight Loss Diets; POUNDS Lost, Preventing Overweight Using Novel Dietary Strategies; PREDIMED, Prevención con Diet Mediterránea; SD, standard deviation.

**Table 2:** Interaction of genotype and fat intake on weight loss.

Gene	SNP	Study Name	Sample Size	Study Population of Interaction Term	Time Point of Weight Measurement	Results (*p*-Value)	Reference
ADAMTS9	rs6795735	NUGENOB	559–580	All participants	10 weeks	0.2 ^1,5,21^	[33]
ADCY3	rs10182181	Obekit	101		16 weeks	*p* = 0.02 ^2,6,21^, *p* = 0.04 ^2,6,22^ Additive model: carriers of the GG (minor allele G) genotype greater weight loss with low-fat diet than carriers of the AG or AA genotypes Co-dominant model: carriers of the GG and AA genotype less weight loss with low-fat diet than carriers of the AG genotype	[35]
ADIPOQ	rs266729	NUGENOB	642		10 weeks	0.029 ^2,7,23^ Carriers of the GG and GC (minor allele G) genotype greater weight loss on high-fat diet than carriers of the CC genotype; Carriers of the GC (minor allele G) genotype greater weight loss on low-fat diet than carriers of the CC genotype	[32]
	rs2241766					0.18 ^2,7,23^	
	rs1501299					0.14 ^2,7,23^	
	rs17300539					0.07 ^2,7,23^	
ADRB2	rs1042713	Obekit	107		4 months	0.71 ^3,8,23^	[36]
	rs1042714					0.86 ^3,8,23^	
AMY1-AMY2	rs11185098	POUNDS Lost	692	Whites + Blacks	2 years	n. s. ^2,9,21^	[40]
APOA5	rs964184		734	All participants		n. s. ^2,10,21^	[50]
CART	rs7379701 *	NUGENOB	642		10 weeks	0.36 ^2,7,23^	[32]
	rs6453132 *					0.10 ^2,7,23^	
	rs17358216					0.72 ^2,7,23^	
	rs5868607					0.68 ^2,7,23^	
CD36	rs2232169					0.42 ^2,7,23^	
CETP	rs3764261	POUNDS Lost + DIRECT	723 + 171	Pooled	2 years	n. s. ^2,11,23^	[45]
CTNNBL1	rs9939609	NUGENOB	559–580	All participants	10 weeks	0.7 ^1,5,21^	[33]
CYP2R1	rs10741657	POUNDS Lost	732		6 months	0.22 ^2,12,21^	[46]
					2 years	0.02 ^2,12,21^ Carriers of the AA (minor allele A) genotype less weight loss with low-fat diet than carriers of the AG or GG genotype	
			576	Whites	6 months	n. s. ^2,11,21^	
					2 years	< 0.05 ^2,11,21^ Carriers of the AA (minor allele A) genotype less weight loss with low-fat diet than carriers of the AG or GG genotype	
DHCR7	rs12785878		732	All participants	6 months	0.80 ^2,12,21^	
					2 years	0.22 ^2,12,21^	
			584	Whites	6 months	n. s. ^2,10,21^	
					2 years	n. s. ^2,10,21^	
DNM3-PIGC	rs1011731	NUGENOB	559–580	All participants	10 weeks	0.2 ^1,5,21^	[33]
ENPP1	rs1799774		642			0.62 ^2,7,23^	[32]
	rs1044498					0.85 ^2,7,23^	
	rs7754561					0.13 ^2,7,23^	
FANCL	rs887912		559–580			0.8 ^1,5,21^	[33]
FOXC2	rs34221221		642			0.75 ^2,7,23^	[32]
FTO	rs9939609	No acronym	188		4 weeks	0.87 ^3,13,23^	[53]
		NUGENOB	734		10 weeks	0.55 ^2,7,24^	[30]
GAD2	rs928197 *		642			0.56 ^2,7,23^	[32]
	rs992990					0.78 ^2,7,23^	
	rs2236418 *					0.88 ^2,7,23^	
GC	rs2282679	POUNDS Lost	732		6 months	0.17 ^2,12,21^	[46]
					2 years	0.08 ^2,12,21^	
			576	Whites	6 months	n. s. ^2,10,21^	
					2 years	n. s. ^2,10,21^	
GHRL	rs696217	NUGENOB	642	All participants	10 weeks	0.33 ^2,7,23^	[32]
GHSR	rs2232169					0.35 ^2,7,23^	
GIPR	rs2287019	POUNDS Lost	737		6 months	0.08 ^2,14,21^	[43]
					2 years	n. s. ^2,14,21^	
			590	Whites	6 months	0.18^2,13,21^	
					2 years	n. s.^2,10,21^	
GPRC5B	rs12444979	NUGENOB	559–580	All participants	10 weeks	0.046^1,5,21^	[33]
HNF1A	rs7957197	POUNDS Lost	722		6 months	0.006 ^2,12,23^ Carriers of the TT and AT (minor allele T) genotype greater weight loss with high-fat diet than carriers of the AA genotype	[41]
			575	Whites		0.001 ^2,15,23^ Carriers of the TT and AT (minor allele T) genotype greater weight loss with high-fat diet than carriers of the AA genotype	
		DIRECT	171	All participants		0.03 ^2,16,23^ Carriers of the TT and AT (minor allele T) genotype greater weight loss with high-fat diet than carriers of the AA genotype	
		POUNDS Lost + DIRECT	722 + 171	Pooled		0.001 ^2,12,23^ Carriers of the TT and AT (minor allele T) genotype greater weight loss with high-fat diet than carriers of the AA genotype	
		POUNDS Lost	722	All participants	2 years	n. s. ^2,10,23^	
		DIRECT	171			n. s. ^2,10,23^	
		POUNDS Lost + DIRECT		Pooled		n. s. ^2,10,23^	
HSD11B1	rs846919	NUGENOB	642	All participants	10 weeks	0.49 ^2,7,23^	[32]
IGF2	rs3168310 *					0.34 ^2,7,23^	
	rs680 *					0.59 ^2,7,23^	
	rs3842759					0.77 ^2,7,23^	
IL6	rs1800795	PREDIMED	737		3 years	0.028 ^3,17,25^ Carriers of the CC (minor allele C) genotype greater weight loss on Mediterranean diet with olive oil than carriers of the CG and GG genotype	[52]
			480	Non-diabetics		0.007 ^3,10,25^ Carriers of the CC (minor allele C) genotype greater weight loss on Mediterranean diet with olive oil than carriers of the CG and GG genotype	
			257	Diabetics		n. s. ^3,10,25^	
		NUGENOB	642	All participants	10 weeks	0.60 ^2,7,23^	[32]
KCNJ11	rs5219					0.10 ^2,7,23^	
LEPROTL1	−2625 C > T					0.12 ^2,7,23^	
LIPC	rs6082					0.42 ^2,7,23^	
	rs1800588 *					0.67 ^2,7,23^	
	rs2070895 *	POUNDS Lost	734		2 years	n. s. ^2,15,21^	[48]
LRRN6C	rs10968576	NUGENOB	559–580		10 weeks	0.1 ^1,5,21^	[33]
LY86	rs1294421					0.6 ^1,5,21^	
MAF	rs1424233					0.1 ^1,5,21^	
MAP2K5	rs2241423					0.3 ^1,5,21^	
MC3R	rs6024728		760			0.89 ^2,7,21^	[31]
	rs6014646					0.48 ^2,7,21^	
	rs6024730					0.57 ^2,7,21^	
	rs6024731					0.72 ^2,7,21^	
	rs11697509					0.20 ^2,7,21^	
	rs6127698					0.81 ^2,7,21^	
	rs3746619 *					0.81 ^2,7,21^	
	rs3827103 *					0.90 ^2,7,21^	
	rs1543873					0.42 ^2,7,21^	
	rs6099058					0.80 ^2,7,21^	
MC4R	rs12970134		559–580			0.4 ^1,5,21^	[33]
MKKS	rs1547		642			0.47 ^2,7,23^	[32]
MTIF3	rs4771122		559–580			0.3 ^1,5,21^	[33]
MTNR1B	rs10830963	POUNDS Lost	575	Whites	6 months	< 0.05 ^2,10,21^ Carriers of the GG (minor allele G) genotype greater weight loss with low-fat diet than carriers of the GC or CC genotype	[37]
			722	All participants		0.01 ^2,12,21^ Carriers of the GG (minor allele G) genotype greater weight loss with low-fat diet than carriers of the GC or CC genotype	
					2 years	0.19 ^2,12,21^	
NFATC2IP	rs11150675		692			0.005 ^2,18,25^ Carriers of the AA (minor allele A) genotype less weight loss with low-fat diet than carriers of the AG and GG genotype	[47]
NPC1	rs1805081	NUGENOB	559–580		10 weeks	0.2 ^1,5,21^	[33]
NPY	rs16147	POUNDS Lost	723		2 years	0.464 ^2,15,21^	[51]
			575	Whites		n. s. ^2,10,21^	
			264	Hypertensive		0.688 ^2,15,21^	
			459	Non-hypertensive		0.547 ^2,15,21^	
PCSK1	rs6235	NUGENOB	642	All participants	10 weeks	0.76 ^2,7,23^	[32]
PPARG2	rs1801282 *	No acronym	1236		Different between participants	0.001 ^4,19,23^ Carriers of the GG and GC (minor allele G) genotype less weight loss (% of baseline weight) on high-fat diet than carriers of the CC genotype	[54]
		NUGENOB	642		10 weeks	0.88 ^2,7,23^	[32]
	rs3856806					0.45 ^2,7,23^	
	rs7649970 *					0.87 ^2,7,23^	
PPARG3	rs10865710					0.76 ^2,7,23^	
PPARGC1A	rs8192678					0.39 ^2,7,23^	
	rs2932963					0.36 ^2,7,23^	
PPM1K	rs1440581		757			0.94 ^2,7,21^	[29]
		POUNDS Lost	734		6 months	0.002 ^2,15,21^ Carriers of the CC (minor allele C) genotype less weight loss with high-fat diet than carriers of the CT or TT genotype	[49]
					2 years	0.008 ^2,15,21^ Carriers of the CC (minor allele C) genotype less weight loss with high-fat diet than carriers of the CT or TT genotype	
			587	Whites	6 months	0.02 ^2,16,21^ Carriers of the CC (minor allele C) genotype less weight loss with high-fat diet than carriers of the CT or TT genotype	
					2 years	0.01^2,16,21^ Carriers of the CC (minor allele C) genotype less weight loss with high-fat diet than carriers of the CT or TT genotype	
RSPO3	rs9491696	NUGENOB	559–580	All participants	10 weeks	0.5 ^1,5,21^	[33]
SERPINE1	rs1799889		642			0.29 ^2,7,23^	[32]
SLC39A8	rs13107325		559–580			0.8 ^1,5,21^	[33]
SLC6A14	rs2011162		481	Women		0.06 ^2,7,23^	[32]
			161	Men		0.78 ^2,7,23^	
SREBF1	17 C > G		642	All participants		0.20 ^2,7,23^	
TCF7L2	rs7903146 *		739			0.023 ^2,7,25^ Carriers of the TT (minor allele T) genotype less weight loss on high-fat diet than carriers of the TC and CC genotype	[17]
		POUNDS Lost	588	Whites	6 months	0.28 ^2,7,21^	[42]
					2 years	0.692 ^2,7,21^	
	rs12255372 *		591		6 months	0.057 ^2,7,21^	
					2 years	0.517 ^2,7,21^	
TFAP2B	rs987237	DiOGenes	640	All participants	8 weeks	0.4 ^1,10,21^	[33]
		NUGENOB	559–580		10 weeks	0.03 ^1,5,21^	
TNFα	rs1800629		642			0.04 ^2,7,23^ Carriers of the AA (minor allele A) genotype greater weight loss on a high-fat diet than carriers of the GG genotype; Carriers of the AG genotype greater weight loss on low-fat diet than carriers of the GG genotype	[32]
UCP2	rs6593669					0.25 ^2,7,23^	
UCP3	rs1900849					0.86 ^2,7,23^	
VEGFA	rs6905288		559–580			0.4 ^1,7,23^	[33]
	rs1358980		707			0.26 ^2,13,26^	[34]
			174	Men		0.58 ^2,20,26^	
			533	Women		0.06 ^2,20,26^	
WAC	rs2807761		642	All participants		0.17 ^2,7,23^	[32]
ZNF608	rs4836133		559–580			0.9 ^1,5,21^	[33]

ADAMTS9, a disintegrin-like and metallopeptidase (reprolysin type) with thrombospondin type 1 motif, 9; ADCY3, adenylate cyclase 3; ADIPOQ, adiponectin, C1Q and collagen domain-containing; ADRB2, adrenoceptor beta 2; AMY1-AMY2, amylase alpha 1A-amylase alpha 2A/B; APOA5, apolipoprotein A5; CART, cocaine- and amphetamine-regulated transcript; CD36, CD36 antigen; CETP, cholesteryl ester transfer protein; CTNNBL1, catenin beta like 1; CYP2R1, cytochrome P450 family 2 subfamily R member; DHCR7, 7-dehydrocholesterol reductase; DiOGenes, Diet, Obesity, and Genes; DIRECT, Dietary Intervention Randomized Controlled Trial; DNM3-PIGC, dynamin 3-phosphatidylinositol glycan anchor biosynthesis class C; ENPP1, ectonucleotide pyrophosphatase/phosphodiesterase 1; FANCL, Fanconi anemia complementation group L; FOXC2, forkhead box C2; FTO, fat mass and obesity-associated; GAD2, glutamate decarboxylase 2; GC, GC vitamin D-binding protein; GHRL, ghrelin; GHSR, growth hormone secretagogue receptor; GIPR, gastric inhibitory polypeptide receptor; GPRC5B, G protein-coupled receptor class C group 5 member B; HNF1A, HNF1 homeobox A; HSD11B1, hydroxysteroid 11-beta dehydrogenase 1; IGF2, insulin-like growth factor 2; IL6, interleukin 6; KCNJ11, potassium inwardly rectifying channel subfamily J member 11; LEPROTL1, leptin receptor overlapping transcript-like 1; LIPC, hepatic lipase C; LRRN6C, leucine rich repeat and immunoglobulin domain-containing 2; LY86, lymphocyte antigen 86; MAF, MAF basic leucine zipper domain transcription factor; MAP2K5, mitogen-activated protein kinase kinase 5; MC3R, melanocortin 3 receptor; MC4R, melanocortin 4 receptor; MKKS, McKusick–Kaufman syndrome; MTIF3, mitochondrial translational initiation factor 3; MTNR1B, melatonin receptor 1B; NFATC2IP, nuclear factor of activated T cells 2-interacting protein; NPC1, Niemann-Pick disease, type C1 intracellular cholesterol transporter 1; NPY, neuropeptide Y; n. s., not significant; NUGENOB, Nutrient-Gene Interactions in Human Obesity: Implications for Dietary Guidelines; Obekit, Development of Nutrigenetic Test for Personalized Prescription of Body Weight Loss Diets; PCSK1, proprotein convertase subtilisin/kexin type 1; POUNDS Lost, Preventing Overweight Using Novel Dietary Strategies; PPARG2, peroxisome proliferator-activated receptor gamma isoform 2; PPARG3, peroxisome proliferator-activated receptor gamma isoform 3; PPARGC1A, peroxisome proliferator-activated receptor gamma coactivator 1 alpha; PPM1K, protein phosphatase, Mg2+/Mn2+-dependent 1K; PREDIMED, Prevención con Diet Mediterránea; RSPO3, R-spondin 3; SERPINE1, serine proteinase inhibitor 1; SLC39A8, solute carrier family 39 member 8; SLC6A14, solute carrier family 6 member 14; SNP, single nucleotide polymorphism; SREBF1, sterol regulatory element-binding transcription factor 1; TCF7L2, transcription factor 7-like 2; TFAP2B, transcription factor activating enhancer binding protein 2 beta; TNFα, tumor necrosis factor alpha; UCP2, uncoupling protein 2; UCP3, uncoupling protein 3; VEGFA, vascular endothelial growth factor A; WAC, WW domain-containing adaptor with coiled-coil; ZNF608, zinc finger protein 608. * SNPs within this gene locus are in high linkage disequilibrium (*r*^2^ > 0.8). ^1^ Interaction term on weight loss in kg for genotype and fat intake in dietetic change (difference in initial and end-of-intervention percentage intake of fat). ^2^ Interaction term on weight loss in kg for genotype and fat intake in energy %. ^3^ Interaction term on weight loss in kg for genotype and Mediterranean diet. ^4^ Interaction term on weight loss in kg for genotype and fat intake according to the intake of the population (below or above the median). ^5^ Adjusted for age, sex, baseline weight, baseline weight x sex, center, genotype, fat intake. ^6^ Adjusted for age, sex, baseline weight. ^7^ Adjusted for age, sex, baseline weight, center. ^8^ Adjusted for age, sex, lipid-lowering medications. ^9^ Adjusted for age, sex, ethnicity, body mass index, weight at baseline, fasting glucose concentration, medication use at baseline. ^10^ No information about adjustment. ^11^ Adjusted for age, sex, ethnicity, baseline body mass index, lipid-lowering medication use. ^12^ Adjusted for age, sex, ethnicity, baseline weight. ^13^ Adjusted for age, sex. ^14^ Adjusted for age, sex, ethnicity. ^15^ Adjusted for age, sex, ethnicity, baseline body mass index. ^16^ Adjusted for age, sex, baseline body mass index. ^17^ Adjusted for age, sex, baseline body mass index, diabetes. ^18^ Adjusted for age, sex, ethnicity, baseline body mass index, smoking status, intervention time. ^19^ Adjusted for age, sex, clinic. ^20^ Adjusted for age. ^21^ Additive genetic model. ^22^ Co-dominant genetic model. ^23^ Dominant genetic model. ^24^ No assumption of genetic model. ^25^ Recessive genetic model. ^26^ No information about genetic model.

**Table 3:** Interaction of genotype and carbohydrate intake on weight loss.

Gene	SNP	Sample Size	Study Population of Interaction Term	Time Point of Weight Measurement	Results (*p*-Value) ^1^	Reference
FGF21	rs838147	715	All participants	2 years	0.07 ^2,5^	[38]
		573	Whites		n. s. ^3,5^	
IRS1	rs2943641	738	All participants	6 months	*p* = 0.037 ^2,5^; *p* = 0.058 ^2,6^ Additive model: carriers of the TT (minor allele T) genotype greater weight loss with the highest-carbohydrate diet than carriers of the TC or CC genotype.	[44]
				2 years	*p* = 0.84 ^2,5^; *p* = 0.59 ^2,6^	
		591	Whites	6 months	*p* < 0.05 ^3,5^; *p* < 0.05 ^3,6^ Additive model: carriers of the TT (minor allele T) genotype greater weight loss with the highest-carbohydrate diet than carriers of the TC or CC genotype.	
				2 years	n. s. ^3,5^, n. s. ^3,6^	
TCF7L2	rs7903146 *	588		6 months	0.811 ^4,5^	[42]
				2 years	0.948 ^4,5^	
	rs12255372 *	591		6 months	0.21 ^4,5^	
				2 years	0.403 ^4,5^	

FGF21, fibroblast growth factor 21; IRS1, insulin receptor substrate 1; n. s., not significant; SNP, single nucleotide polymorphism; TCF7L2, transcription factor 7-like 2. * SNPs are in high linkage disequilibrium (*r*^2^ > 0.8). ^1^ interaction term on weight loss in kg for genotype and carbohydrate intake in energy %. All articles investigated data from the Preventing Overweight Using Novel Dietary Strategies (POUNDS Lost) trial. ^2^ Adjusted for age, sex, baseline weight, ethnicity. ^3^ No information about adjustment. ^4^ Adjusted for age, sex, center, baseline weight. ^5^ Additive genetic model. ^6^ Dominant genetic model.

**Table 4:** Interaction of genotype and protein intake on weight loss.

Gene	SNP	Sample Size	Study Population of Interaction Term	Time Point of Weight Measurement	Results (*p*-Value) ^1^	Reference
AMY1-AMY2	rs11185098	692	All participants	2 years	n. s. ^2^	[40]
CYP2R1	rs10741657	732		6 months	0.48 ^3^	[46]
				2 years	0.19 ^3^	
		576	Whites	6 months	n. s. ^8^	
				2 years	n. s. ^8^	
DHCR7	rs12785878	732	All participants	6 months	0.25 ^3^	
				2 years	0.52 ^3^	
		584	Whites	6 months	n. s. ^8^	
				2 years	n. s. ^8^	
GC	rs2282679	732	All participants	6 months	0.68 ^3^	
				2 years	0.41 ^3^	
		576	Whites	6 months	n. s. ^8^	
				2 years	n. s. ^8^	
GIPR	rs2287019	737	All participants	6 months	> 0.35 ^4^	[43]
				2 years		
		590	Whites	6 months	> 0.35 ^9^	
				2 years		
LCT	rs4988235	583			n. s. ^5^	[39]
NPY	rs16147	723	All participants		n. s. ^6^	[51]
		575	Whites		n. s. ^8^	
PPM1K	rs1440581	734	All participants	6 months	> 0.05 ^6^	[49]
				2 years		
		587	Whites	6 months	> 0.05 ^10^	
				2 years		
TCF7L2	rs7903146 *	588		6 months	0.906 ^7^	[42]
				2 years	0.515 ^7^	
	rs12255372 *	591		6 months	0.746 ^7^	
				2 years	0.328 ^7^	

AMY1-AMY2, amylase alpha 1A-amylase alpha 2A/B; CYP2R1, cytochrome P450 family 2 subfamily R member; DHCR7, 7-dehydrocholesterol reductase; GC, GC vitamin D-binding protein; GIPR, gastric inhibitory polypeptide receptor; LCT, lactase; NPY, neuropeptide Y; n. s., not significant; PPM1K, protein phosphatase, Mg2+/Mn2+-dependent 1K; SNP, single nucleotide polymorphism; TCF7L2, transcription factor 7-like 2. * SNPs are in high linkage disequilibrium (*r*^2^ > 0.8). ^1^ Interaction term on weight loss in kg for genotype and protein intake in energy %. All interactions were analyzed with an additive genetic model and all articles investigated data from the Preventing Overweight Using Novel Dietary Strategies (POUNDS Lost) trial. ^2^ Adjusted for age, sex, ethnicity, body mass index, baseline weight, fasting glucose concentration, medication use at baseline. ^3^ Adjusted for age, sex, ethnicity, baseline weight. ^4^ Adjusted for age, sex, ethnicity. ^5^ Adjusted for age, sex, body mass index, baseline weight. ^6^ Adjusted for age, sex, ethnicity, baseline body mass index. ^7^ Adjusted for age, sex, center, baseline weight. ^8^ No information about adjustment. ^9^ Adjusted for age, sex. ^10^ Adjusted for age, sex, baseline body mass index.
